# PACAP-38 induces neuronal differentiation of human SH-SY5Y neuroblastoma cells via cAMP-mediated activation of ERK and p38 MAP kinases^1^

**DOI:** 10.1111/j.1471-4159.2007.05018.x

**Published:** 2008-01

**Authors:** T K Monaghan, C J MacKenzie, R Plevin, E M Lutz

**Affiliations:** Strathclyde Institute of Pharmacy and Biomedical Sciences, Royal College Glasgow, UK

**Keywords:** cAMP-dependent, Epac, neuritogenesis, PAC_1_ receptor, PACAP, VIP

## Abstract

The intracellular signaling pathways mediating the neurotrophic actions of pituitary adenylate cyclase-activating polypeptide (PACAP) were investigated in human neuroblastoma SH-SY5Y cells. Previously, we showed that SH-SY5Y cells express the PAC_1_ and VIP/PACAP receptor type 2 (VPAC_2_) receptors, and that the robust cAMP production in response to PACAP and vasoactive intestinal peptide (VIP) was mediated by PAC_1_ receptors (Lutz *et al.* 2006). Here, we investigated the ability of PACAP-38 to differentiate SH-SY5Y cells by measuring morphological changes and the expression of neuronal markers. PACAP-38 caused a concentration-dependent increase in the number of neurite-bearing cells and an up-regulation in the expression of the neuronal proteins Bcl-2, growth-associated protein-43 (GAP-43) and choline acetyltransferase: VIP was less effective than PACAP-38 and the VPAC_2_ receptor-specific agonist, Ro 25-1553, had no effect. The effects of PACAP-38 and VIP were blocked by the PAC_1_ receptor antagonist, PACAP6-38. As observed with PACAP-38, the adenylyl cyclase activator, forskolin, also induced an increase in the number of neurite-bearing cells and an up-regulation in the expression of Bcl-2 and GAP-43. PACAP-induced differentiation was prevented by the adenylyl cyclase inhibitor, 2′,5′-dideoxyadenosine (DDA), but not the protein kinase A (PKA) inhibitor, H89, or by siRNA-mediated knock-down of the PKA catalytic subunit. PACAP-38 and forskolin stimulated the activation of extracellular signal-regulated kinase (ERK), mitogen-activated protein kinase (MAP; p38 MAP kinase) and c-Jun N-terminal kinase (JNK). PACAP-induced neuritogenesis was blocked by the MEK1 inhibitor PD98059 and partially by the p38 MAP kinase inhibitor SB203580. Activation of exchange protein directly activated by cAMP (Epac) partially mimicked the effects of PACAP-38, and led to the phosphorylation of ERK but not p38 MAP kinase. These results provide evidence that the neurotrophic effects of PACAP-38 on human SH-SY5Y neuroblastoma cells are mediated by the PAC_1_ receptor through a cAMP-dependent but PKA-independent mechanism, and furthermore suggest that this involves Epac-dependent activation of ERK as well as activation of the p38 MAP kinase signaling pathway.

The 38-amino acid pituitary adenylate cyclase (AC) activating polypeptide (PACAP) is an important neuropeptide and neuroendocrine hormone that was first isolated from ovine hypothalamus extracts because of its potent ability to activate AC (Miyata *et al.* 1989). There are several actions associated with PACAP in both the developing and mature nervous systems (reviewed by Vaudry *et al.* 2000). PACAP is a well-documented neurotrophic factor, regulating neuronal survival, neurotransmitter phenotype, axon growth and growth cone attraction (DiCicco-Bloom and Deutsch 1992; Gonzalez *et al.* 1997; Villalba *et al.* 1997; Vaudry *et al.* 1999; Borba *et al.* 2005; Dejda *et al.* 2005; Falluel-Morel *et al.* 2005). In the mature nervous system, PACAP has been shown to act as a neurotransmitter and neuromodulator and has also been shown to increase cell survival after neuronal trauma (Harrington *et al.* 1999; Inoue *et al.* 2000; Onoue *et al.* 2002; Farkas *et al.* 2004; Tamas *et al.* 2006). PACAP expression is up-regulated at sites of neuronal injury (Zhang *et al.* 1995, 1996; Moller *et al.* 1997; Boeshore *et al.* 2004) where it may help prevent cell death and promote neuronal regeneration.

The actions of PACAP are mediated through three G protein-coupled receptors (GPCRs), the VPAC_1_, VPAC_2_, and PAC_1_ receptors (Zhou *et al.* 2002), which are members of the Group II secretin receptor family (Harmar 2001). PACAP and the structurally related neuropeptide, vasoactive intestinal peptide (VIP), have similar potencies at the VPAC receptors (Laburthe *et al.* 2002), whereas PACAP is ≥100-fold more potent than VIP at the PAC_1_ receptor (Lutz *et al.* 2006). Similar to other Group II GPCRs, the VPAC and PAC_1_ receptors couple to the activation of AC (Harmar and Lutz 1994). In addition, VPAC and PAC_1_ receptors differentially couple to the activation of phospholipase C and phospholipase D (Spengler *et al.* 1993; Langer *et al.* 2001; MacKenzie *et al.* 2001; McCulloch *et al.* 2001).

Although PACAP has long been known to be involved in neuronal development and regeneration, very little is known about the signal transduction pathways which mediate the neurotrophic effects of PACAP on human neuronal cells. The human neuroblastoma cell line SH-SY5Y is a well-characterized model for neuronal differentiation (Påhlman *et al.* 1990). These cells have been shown to respond to various differentiation factors, such as retinoic acid (RA), staurosporine, or brain-derived neurotrophic factor by an increase in neurite outgrowths and up-regulation of the expression of markers of neuronal differentiation including Bcl-2 and GAP-43 (Leli *et al.* 1992; Hanada *et al.* 1993; Encinas *et al.* 1999; Feng and Porter 1999; Jamsa *et al.* 2004; Pan *et al.* 2005). Recently, it has been shown that PACAP induces neurite outgrowths and increased expression of neuronal cytoskeletal proteins in SH-SY5Y cells (Héraud *et al.* 2004), however, the signal transduction mechanisms underlying this were not determined. We have shown previously that SH-SY5Y cells express PAC_1_ and VPAC_2_ receptors and that PAC_1_ receptors mediate the activation of cAMP production in these cells by PACAP and VIP (Lutz *et al.* 2006). Here we have investigated PAC_1_ receptor-mediated activation of cAMP production in promoting the differentiation of SH-SY5Y cells by PACAP.

## Materials and methods

### Materials

Tissue culture media were obtained from Sigma–Aldrich (Poole, UK), and animal sera from Biowest Ltd. (Ringmer, UK). The SH-SY5Y cell line was obtained from ECACC (Salisbury, UK). Peptides, all-*trans* retinoic acid, phorbol-12-myristate-13-acetate (PMA) and enzyme inhibitors were supplied by Merck Biosciences (UK) Ltd. (Nottingham, UK), except for PD98059 which was obtained from Promega (Southampton, UK). The selective VPAC_2_ receptor agonist, Ro 25-1553, was obtained from Dr. Patrick Robberecht, Université Libre de Bruxelles, Brussels, Belgium. The exchange protein directly activated by cAMP (Epac) activator, 8-(4-chlorophenylthio)-2′O-methyladenosine 3′,5′-cyclic monophosphate sodium salt was obtained from Sigma–Aldrich. Antibodies against Bcl-2, ERK, phosphoERK, GAP-43, tyrosine hydroxylase (TH) and GAPDH were obtained from Santa Cruz Biotechnology (Autogen Bioclear UK Ltd., Calne, UK), all other antibodies were obtained from Biosource (Paisley, UK). Standard laboratory chemicals of Analar grade were obtained from Sigma–Aldrich or BDH Chemicals Ltd. (Poole, UK).

### Cell culture

SH-SY5Y cells were maintained at 37°C in a humidified atmosphere of 5% CO_2_ and 95% air and cultured in a 1 : 1 mixture of Ham’s F12 and Eagle’s minimum essential medium supplemented with non-essential amino acids, l-glutamine, sodium pyruvate, 100 U/mL each of penicillin and streptomycin, and 5% fetal bovine serum (FBS). Medium was changed every fourth day. All the cells used in this study were used at a low passage number (<20).

### cAMP assay

The production of cAMP was measured by the β-galactosidase enzyme fragment complementation-based HitHunter cAMP XS+ chemiluminesence assay (DiscoveRx, Birmingham, UK) according to the manufacturer’s instructions. Briefly, SH-SY5Y cells at ∼80% confluency in 75 cm^2^ flasks were quiesced for 2 h in OptiMEM (Invitrogen Ltd., Paisley, UK) before being scraped into phosphate-buffered saline and counted. Cells were centrifuged at 400 *g* for 5 mins and resuspended in MEM containing 0.25% bovine serum albumin (BSA; Sigma-Aldrich) and 500 μmol/L 3-isobutyl-1-methylxanthine for 15 min. Cells were assayed in suspension by putting 10^4^ cells per well into a 96-well plate before stimulating with varying concentrations of ligand. The assay was stopped after 15 min by lysing cells and luminescence was detected using a LUMIstar Galaxy plate reader (BMG Labtech Ltd., Aylesbury, Bucks, UK). In addition, SH-SY5Y cells plated into 24-well plates were pre-labeled overnight with [^3^H]-adenine and [^3^H]cAMP production measured as described by [Wong, 1994 #36]. [^3^H]ATP/ADP and [^3^H]cAMP were isolated from each sample by sequential chromatography on anion exchange and alumina columns and the amount of radioactivity of the flow through (representing [^3^H]ATP+[^3^H]ADP) and eluate (representing [^3^H]cAMP) measured by scintillation counting. The levels of cAMP production were calculated as the ratio of [[^3^H]cAMP]/([[^3^H]cAMP] + [[^3^H]ATP+[^3^H]ADP]).

### MAP kinase phosphorylation assay

SH-SY5Y cells were seeded into 12-well plates at a density of 3 × 10^6^ cells per well and cultured for 48 h, after which the medium was replaced with low-serum medium containing 0.2% FBS and incubated overnight. Medium was replaced with fresh low-serum medium for 2 h before agonists were added, inhibitors were added 45 min before adding agonists. Stimulation was halted by placing plates on ice and cells harvested by preparing whole cell extracts on ice after aspirating the medium and adding lysis buffer (250 mmol/L Tris, pH 6.8, 14 mmol/L sodium pyrophosphate, 20 mmol/L ethylenediaminetetraacetic acid (EDTA), 35 mmol/L sodium dodecyl sulphate as described previously (McLees *et al.* 1995). Cells scraped into lysis buffer were homogenized by passing through a 21G needle and protein concentration quantified using a standard Coomassie-based Bradford assay.

### Differentiation of SH-SY5Y cells

SH-SY5Y cells were plated into 12-well plates at a density of 10^5^ cells per well and cultured for 48 h. Before starting treatments, the cells were incubated for 16–24 h in low-serum medium containing 0.2% FBS, then photographed under a 40× objective lens using a Nikon Coolpix 4500 camera attached to an Olympus CK40 microscope (Microscopes Scales & Servicing, Glasgow, UK). Medium was replaced with fresh low-serum medium and treatments added. Where indicated, inhibitors were added 45 min before the treatment. Micrographs were taken 48 h after the initial treatment and medium-containing treatments were renewed; this was repeated every 48 h for up to 8 days. Cells were harvested as described above.

### Cell morphology analysis

Cell morphology analysis was carried out on captured micrographs of treated cells. For each treatment, the total number of cells and total number of cells with neurites longer than the length of the cell body were manually assessed from at least six micrographs in a blind count. The ratio between the number of neurite-bearing cells and total number of cells was calculated for each treatment, and the fold change over the control was determined. Each experiment was repeated a minimum of three times and variance between datasets determined by anova analysis using GraphPad Prism (GraphPad Software, Inc., San Diego CA, USA). Adobe Photoshop was used to adjust the contrast of the micrograph images.

### SDS–PAGE and western blots

Whole cell extracts were mixed with an equal volume of 2X standard Laemmli buffer, containing 100 mmol/L dithiothreitol, and denatured at 100°C for 5 min. 20 μg of protein was then separated on a 10% sodium dodecyl sulphate–polyacrylamide gel electrophoresis gel and transferred onto Hybond ECL™ nitrocellulose membrane (GE Healthcare, Chalfont St Giles, UK). Membranes were blocked for 2 h at 17°C with 2% BSA in Tris-buffered saline/0.03% Tween-20 (TBST), then incubated overnight with primary antibody as indicated, diluted in TBST containing 0.2% BSA. Following several washes in TBST, membranes were incubated with horseradish peroxidase-conjugated secondary antibody (GE Healthcare) and detected by enhanced chemiluminescence using a Fuji LAS-3000 imager (Raytek Scientific Ltd., Sheffield, UK). Western blots were quantified using Kodak 1D Image Analysis software and given as a ratio of net intensity/band area (pixels).

### siRNA knock-down

Expression of the protein kinase A catalytic subunit (PKAc) was inhibited using the SignalSilence PKAc siRNA kit (Cell Signalling Technology, Hitchin, Hertfordshire, UK) used according to the manufacturer’s instructions. Briefly, 10^5^ cells were transfected with 30 pmoles siRNA oligonucleotide and incubated for 48 h before treatment. Knock-down of PKAc was assessed by western blotting and specificity of RNA interference determined by western blotting for the related protein, protein kinase B.

### Immunocytochemistry

Cells were plated at a density of 10^4^ cells per well onto fibronectin-coated coverslips in 12-well plates and incubated for 48 h. Cells were washed in ice-cold tris-bufferd saline (TBS) and fixed at −20°C in methanol for 10 min before incubating with primary antibody diluted in TBS/1% BSA, as indicated, overnight at 4°C. After several washes in TBS, cells were probed with AlexaFluor conjugated secondary antibody (Invitrogen) and mounted on to slides using Moviol (65 μmol/L polyvinyl alcohol, 80 mmol/L Tris. pH 8.5, 2.5% 1,4-diazobicyclooctane, 17% glycerol). Cells were examined with a Nikon fluorescent microscope and image analysis carried out using the IP Labs 3.7 software (Nikon UK Ltd., Surrey, UK).

### Data analysis

Curve fitting was accomplished using GraphPad Prism (GraphPad Software, Inc., San Diego CA, USA). The data are expressed as mean ± standard error (SE). One- and two-way anova were carried out as appropriate to identify significant differences between treatments.

## Results

### PACAP-38 induces neuritogenesis and the expression of neuronal marker proteins in SH-SY5Y cells

**Fig. 1:**
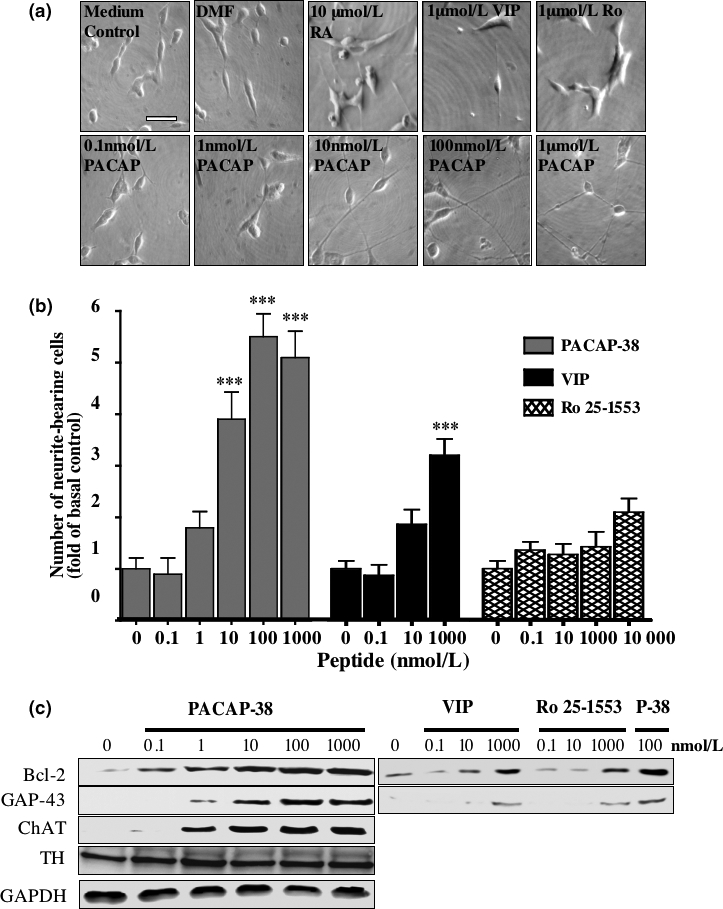
The effect of PACAP on differentiation of SH-SY5Y cells. SH-SY5Y cells were cultured in low serum medium for 24 h before being incubated with increasing concentrations of PACAP-38 (PACAP), retinoic acid (RA), VIP, Ro 25-1553 or dimethylformamide, as indicated. Medium and treatments were replaced after 2 days and micrographs captured after 4 days (a). Micrographs are set to the same scale; the scale bar represents 50 μm. (b) The total number of cells, and the number of cells bearing neurites greater than the length of the cell body were counted and the data expressed as fold of basal control. Experiments were performed in triplicate and one- and two-way ANOVA carried out to determine differences between treatments (***p < 0.001). (c) After capture of micrographs, whole cell extracts were taken and the proteins separated and blotted as detailed in Materials and methods. Antibodies specific for Bcl-2, GAP-43, ChAT, TH and GAPDH were used as indicated. Cell extracts from cells treated with 100 nmol/L PACAP-38 (P-38) were run alongside those from cells treated with VIP and Ro 25-1553 at the indicated concentrations for comparison. Each blot is representative of three independent experiments.

Cells maintained in medium containing 0.2% FBS (low serum medium) were treated with different concentrations of PACAP-38 (0.1 nmol/L to 1 μmol/L) and examined for changes in cell morphology and expression of neuronal differentiation-associated proteins. The number of neurite-bearing cells increased within 2 days of treatment: those treated with 10 nmol/L to 1 μmol/L PACAP-38 displayed a bipolar cell body with long, thin, neurite-like processes by day 4 (Fig. 1a). Cells treated with 10 μmol/L RA also developed long, thin, neurite extensions (Fig. 1a), as shown in previous studies (Påhlman *et al.* 1984), whereas control cells treated with dimethylformamide (vehicle) or maintained in low serum medium alone did not. Since SH-SY5Y cells express PAC_1_ receptor splice variants that are responsive to VIP, as well as the VIP/PACAP-responsive VPAC_2_ receptors (Lutz *et al.* 2006), the effects of VIP and the VPAC_2_ receptor-specific agonist, Ro 25-1553, were tested. VIP was also able to induce the development of neurite-like processes, but required 100-fold higher concentrations than PACAP-38 (Fig. 1a) and only the highest concentration of Ro 25-1553 (10 μmol/L) elicited the development of neurites in a small proportion of cells. Figure 1b shows the increase in neurite-bearing cells on day 4 in response to PACAP-38, VIP, and Ro 25-1553. A concentration-dependent increase was observed, with 100 nmol/L PACAP-38 eliciting the greatest increase (5.6 ± 0.5-fold), compared to 1 μmol/L VIP (3.2 ± 0.4-fold) and 10 μmol/L Ro 25-1553 (2.1 ± 0.3-fold). The number of neurite-bearing cells increased by 8.3 ± 1.3-fold (*p* < 0.001) following 4 day treatment with 10 μmol/L RA.

The effects of various concentrations of PACAP-38, VIP, and Ro 25-1553 on the expression of Bcl-2 and GAP-43 were assessed by western blotting (Fig. 1c). Protein loading for each sample was checked by blotting for the housekeeping gene glyceraldehyde-3-phosphate dehydrogenase (GAPDH). Low concentrations of PACAP-38 were found to increase the expression of Bcl-2 and GAP-43 (0.1 nmol/L and 1 nmol/L, respectively). In comparison, much higher concentrations of VIP were needed to induce expression of these two markers of neuronal differentiation (approximately 10 nmol/L and 1 μmol/L, respectively, Fig. 1c), whereas Ro 25-1553 was even less potent. In addition, the ability of PACAP-38 to affect the neurotransmitter phenotype was determined by examining the expression of choline acetyltransferase (ChAT), a marker for cholinergic neurones, and tyrosine hydroxylase (TH), a marker for noradrenergic neurones. SH-SY5Y cells normally express TH, and the levels of TH were not significantly affected by the 4-day treatment of cells with the varying concentrations of PACAP-38 used in this study (Fig. 1c). However, treatment of SH-SY5Y cells with 1 nmol/L PACAP-38 was sufficient to induce expression of ChAT, the level of expression increasing in a concentration-dependent manner. (Fig. 1c).

### PACAP6-38 inhibits PACAP-38-induced neuronal differentiation of SH-SY5Y cells

**Fig. 2:**
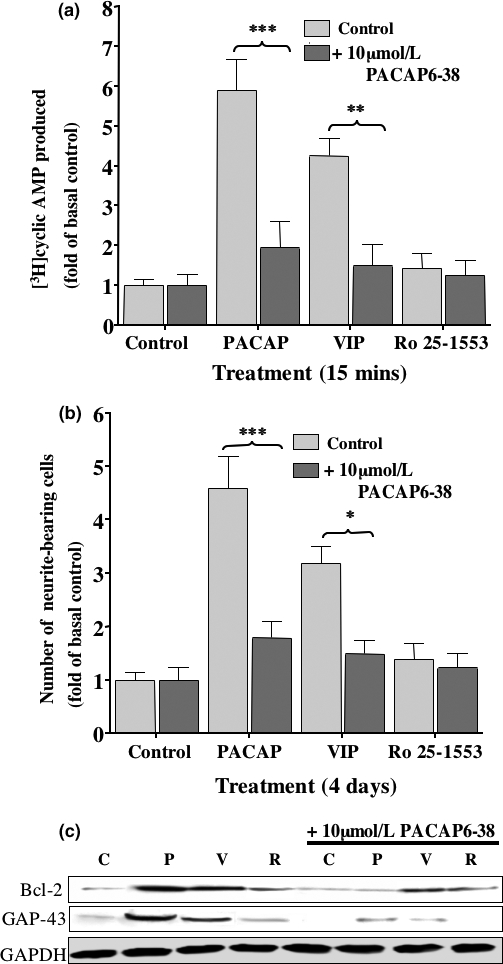
The effects of the PAC_1_ receptor antagonist PACAP6-38 on SH-SY5Y cells. SH-SY5Y cells were maintained in low serum medium as described in Materials and methods. (a) Histogram showing fold change in cAMP levels for cells treated for 15 min with 100 nmol/L PACAP-38, 1 μM VIP or 1 μM Ro 25-1553, in the presence (dark gray bars), or absence (light gray bars) of 10 μM PACAP6-38. The basal level was determined for cells maintained in low serum medium alone. (b) Histogram showing numbers of neurite-bearing cells (fold of basal control) following treatment for 4 days with 100 nmol/L PACAP-38, 1 μM VIP or 1 μM Ro 25-1553, in the presence (dark gray bars) or absence (light gray bars) of 10 μM PACAP6-38. Standard error bars are shown (*n* = 3) and one-way ANOVA performed to identify significant differences between treatments (***p < 0.001, **p < 0.01, *p < 0.05). (c) Western blot analysis of cell extracts using antibodies specific for Bcl-2 and GAP-43 was carried out following the different treatments for 4 days. Protein loading was determined using an antibody specific for GAPDH. Each blot represents three independent experiments. (C = control, *p* = 100 nmol/L PACAP-38, V = 1 μM VIP, R = 1 μmol/L Ro 25-1553).

The PAC_1_ receptor antagonist PACAP6-38 was used in order to determine if PAC_1_ receptors are involved in mediating the differentiating effects of PACAP-38 on SH-SY5Y cells. This inhibitor has also been shown to act at VPAC_2_ receptors (Dickinson *et al.* 1997; Moro *et al.* 1999), however, since Ro 25-1553 did not have a significant effect on neuritogenesis or the expression of Bcl-2 and GAP-43, it seems unlikely that the VPAC_2_ receptor has a significant role in mediating PACAP-38-induced neuronal differentiation of SH-SY5Y cells. The ability of PACAP6-38 to inhibit PACAP-38-, VIP- and Ro 25-1553-stimulated cAMP production was tested in SH-SY5Y cells. Fig. 2a shows that the inhibitor had no effect on basal levels of cAMP, but significantly inhibited 100 nmol/L PACAP-38- and 1 μmol/L VIP-stimulated cAMP production by 81 ± 25% and 85 ± 26%, respectively. Likewise, 4-day treatment with 10 μmol/L PACAP6-38 did not significantly alter the basal number of neurite-bearing cells whereas the 100 nmol/L PACAP-38- or 1 μmol/L VIP-stimulated increase in the number of neurite-bearing cells was significantly reduced, by 78 ± 23% and 77 ± 22%, respectively, following co-treatment with 10 μmol/L PACAP6-38 (Fig. 2b). PACAP-38-induced expression of Bcl-2 and GAP-43 was also inhibited by 10 μmol/L PACAP6-38 (Fig. 2c). Thus, it appears likely that this is mediated principally through the PAC_1_ receptor.

### Production of cAMP is required for PACAP-38-induced neuronal differentiation of SH-SY5Y cells

**Fig. 3:**
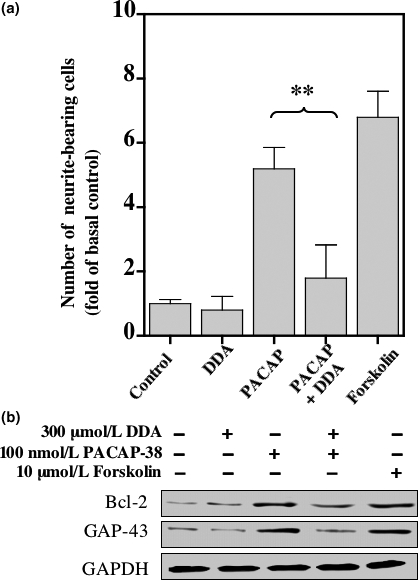
The effects of DDA and Forskolin on differentiation of SH-SY5Y cells. SH-SY5Y cells were maintained in low serum medium as previously described. (a) Histogram showing for the numbers of neurite-bearing cells following treatment with 100 nmol/L PACAP-38 in the presence or absence of 300 μmol/L DDA, or for cells treated with 10 μmol/L forskolin for 4 days. Standard error bars are shown (*n* = 3), one-way ANOVA analysis was carried out to identify significant differences between treatments (**p < 0.01). (b) Western blot analysis of cell extracts using antibodies specific for Bcl-2 and GAP-43 was carried out following the different treatments for 4 days. Protein loading was determined using an antibody specific for GAPDH. Each blot represents three independent experiments.

Treatment of SH-SY5Y cells with cAMP analogues has been shown to induce differentiation (Sánchez *et al.* 2004). Since PACAP elicits a robust activation of cAMP production in SH-SY5Y cells through the PAC_1_ receptor (Lutz *et al.* 2006), the AC inhibitor, DDA, was used in this study to determine if PACAP-38-induced differentiation of SH-SY5Y cells was cAMP-dependent. In SH-SY5Y cells 300 μmol/L DDA inhibited 100 nmol/L PACAP-38-stimulated cAMP production to 25 ± 19% of the PACAP-38-stimulated control (*n* = 3), DDA was calculated to have an IC_50_ of 208 ± 0.3 nmol/L (*n* = 3: data not shown). The AC activator, forskolin, was used for comparison to PACAP-38. Forskolin elicits cAMP production with an EC_50_ of 5.8 ± 0.2 μmol/L and *E*_max_ of 12 ± 1-fold of basal control in SH-SY5Y cells (data not shown). Cells were then maintained in: low serum medium; 300 μmol/L DDA; 100 nmol/L PACAP-38; 300 μmol/L DDA and 100 nmol/L PACAP-38; 10 μmol/L forskolin. Changes in cell morphology and expression of Bcl-2 and GAP-43 were examined after 4 days of treatment. As shown in Fig. 3a, treatment with DDA alone had no effect compared to control cells but inhibits the PACAP-38-induced increase in neurite-bearing cells by approximately 76 ± 36%. Furthermore, DDA had no effect on basal levels of Bcl-2 and GAP-43 expression compared to control cells, but caused a near complete inhibition of their induction by 100 nmol/L PACAP-38 (Fig. 3b). Four day treatment of SH-SY5Y cells maintained in low serum medium with 10 μmol/L forskolin caused an increase in the number of neurite-bearing cells to 6.8 ± 1.0-fold of basal control (Fig. 3a) and induced the expression of Bcl-2 and GAP-43 (Fig. 3b).

### Protein kinase A is not required for PACAP-38-induced neuritogenesis

**Fig. 4:**
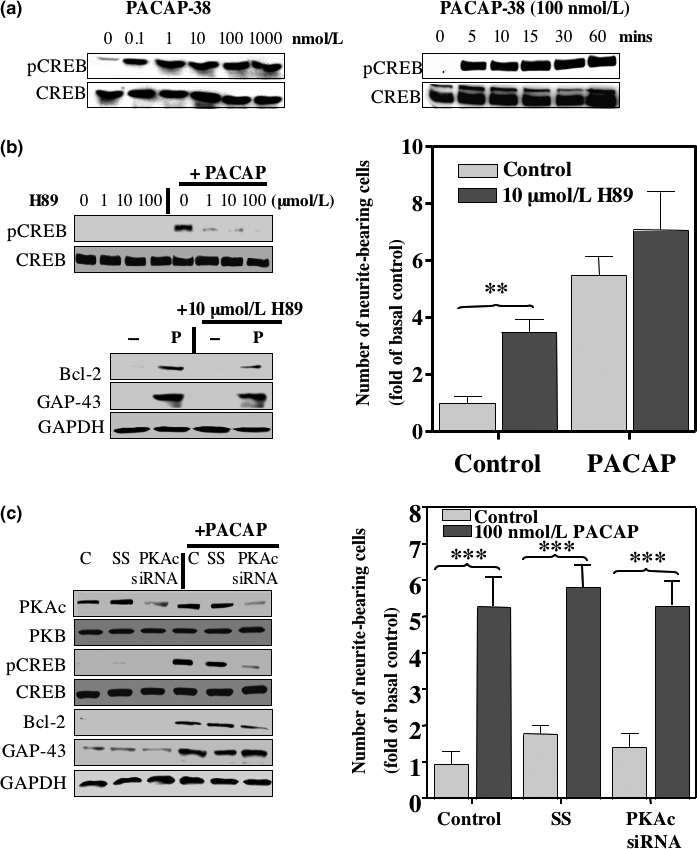
The effects of PKA inhibition on differentiation of SH-SY5Y cells. SH-SY5Y cells were maintained in low serum medium as previously described. (a) Western blots showing changes in CREB phosphorylation following a 15 min. treatment with increasing concentrations of PACAP-38 (left panel) and a time course of 100 nmol/L PACAP-38-induced CREB phosphorylation (right panel). The total CREB protein in each sample is shown in the lower panels. (b) The effects of treating cells with H89 were assessed by Western blot analysis of pCREB levels in whole cell extracts from cells treated with 1, 10 and 100 μmol/L H89 in the presence or absence of 100 nmol/L PACAP-38 for 15 min (upper panel). The levels of total CREB protein were also assessed for each sample. The effect of 10 μmol/L H89 on levels of Bcl-2 and GAP-43 protein expression in cells in the presence or absence of 100 nmol/L PACAP-38 for 4 days are shown in the lower panels. The right panel is a histogram showing numbers of neurite-bearing cells (fold of basal control) following treatment with 10 μmol/L H-89, in the presence (dark gray bars) or absence (light gray bars) of 100 nmol/L PACAP-38. (c) Western blots showing changes in expression of PKAc and PKB 2 days after siRNA transfection to knock-down PKAc expression, and its affect on 100 nmol/L PACAP-38-induced CREB (C = control, SS = scrambled siRNA sequence, PKAc siRNA = specific siRNA sequence targeting the catalytic subunit of PKA). The right panel is a histogram showing numbers of neurite-bearing cells (fold of basal control) following siRNA transfection to knock-down PKAc expression, in the presence (dark gray bars) or absence (light gray bars) of 100 nmol/L PACAP-38. ANOVA analysis was performed to identify significant differences between treatments (***p < 0.001, **p < 0.01). Protein loading in (b) and (c) was determined using an antibody specific for GAPDH. Each blot is representative of three independent experiments.

As protein kinase A (PKA) is one of the major intracellular effectors activated by elevated levels of cAMP, we set out to determine if PKA is required for PACAP-38-mediated differentiation of SH-SY5Y cells. The transcription factor cAMP response element binding protein (CREB) is a downstream target of PKA and was used in this study as a measure of PACAP-38-mediated PKA activation and the effectiveness of PKA inhibitors. Concentrations of PACAP-38 as low as 0.1 nmol/L elicited an increase in pCREB over basal levels (Fig. 4a, upper left panel). For cells treated with 100 nmol/L PACAP-38, pCREB levels were elevated within 5 min and remained elevated for at least 60 min (right panel, Fig. 4a). PACAP-38-stimulated CREB phosphorylation was inhibited by treating cells with the PKA inhibitor H89 at concentrations of 1, 10, and 100 μmol/L (Fig. 4b). Interestingly, treatment of cells with 10 μmol/L H89 in low serum medium for 4 days elicited a significant increase in the number of neurite-bearing cells to 3.5 ± 0.3-fold of basal control and augmented the 100 nmol/L PACAP-38-induced increase (Fig. 4b, right panel), although this augmentation was not significant compared to PACAP-38 alone. The PACAP-38-induced expression of Bcl-2 was partially inhibited by 10 μmol/L H89, however, that of GAP-43 was not affected (Fig. 4b, lower left panel).

Although H89 is widely used as a specific PKA inhibitor, it has been shown to potently inhibit other kinases including Rho kinase/ROCK-II (Davies *et al.* 2000). Inhibition of this kinase rather than PKA by H89 has been shown to lead to the formation of neurite-like extensions in NG108-15 cells (Leemhuis *et al.* 2002). In order to explore further the role of PKA in PACAP-38-induced neurite extensions in SH-SY5Y cells, siRNA was used to knock-down expression of the PKA catalytic subunit. The effectiveness and specificity of this is shown in the top panel of Fig. 4c, where expression of the catalytic subunit of PKA is decreased by treatment with PKAc siRNA, but not with the scrambled siRNA sequence (ss). Expression of the closely related protein kinase B (second panel, Fig. 4c) is not affected by either treatment. Down-regulation of PKAc by siRNA effectively inhibits the increase in pCREB levels in cells treated with 100 nmol/L PACAP-38 (third panel, Fig. 4c), but only partially reduces PACAP-38-induced Bcl-2 expression and does not affect that of GAP-43 (bottom panels, Fig. 4c), as we have observed following H89 treatment (Fig. 4b). However, down-regulation of PKAc had no significant effect on the formation of neurite extensions compared to controls, and did not augment 100 nmol/L PACAP-38-induced increase in neurite-bearing cells compared to control cells (graph, Fig. 4c), as found with H89. Taken together, these results suggest that PKA activity is required for PACAP-38-induced expression of Bcl-2, but not for GAP-43 or for PACAP-38-induced neuritogenesis. Furthermore, they suggest that H89 may be inhibiting another protein kinase in addition to PKA in SH-SY5Y cells, as observed in NG108-15 cells (Leemhuis *et al.* 2002). The identity of this kinase remains to be established.

### PACAP-38-induced neuronal differentiation of SH-SY5Y cells is dependent upon ERK and p38 MAP kinase activity

**Fig. 5:**
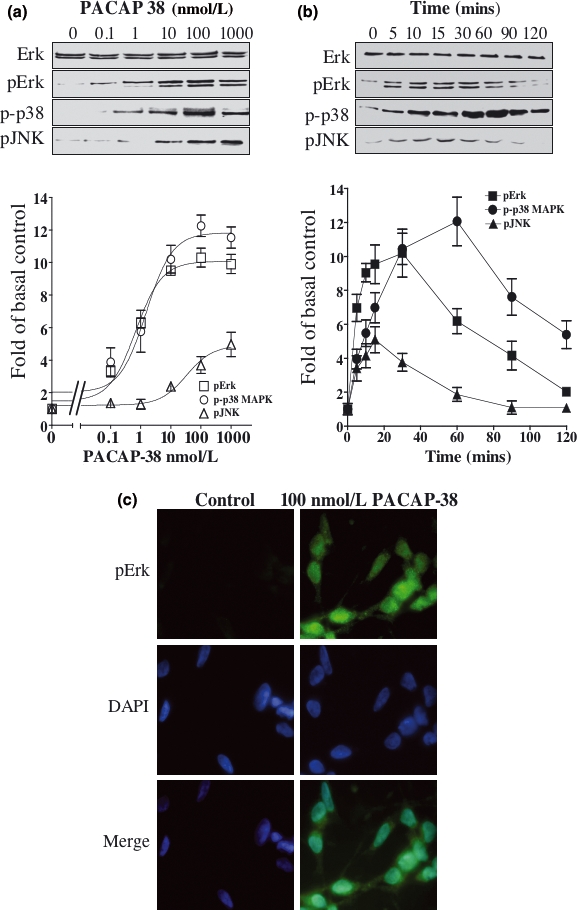
PACAP-38-mediated activation of ERK, p38 MAP kinase and JNK. SH-SY5Y cells were cultured in low serum medium for 24 h and incubated with increasing concentrations of PACAP-38 for 15 min (a) or with 100 nmol/L PACAP-38 for up to 2 h (b). Western blot analysis of whole cell extracts was carried out using antibodies specific for ERK, pERK, p-p38 MAP kinase and pJNK. The increase in phosphorylation levels was determined by densitometry as described in Materials and methods and is graphically represented in the lower section of the figure. Standard error bars are shown (*n* = 5). (c) The cells were incubated with a pERK-specific antibody and its respective fluorescent dye-conjugated secondary antibody (green) following treatment with 100 nmol/L PACAP-38 for 30 min. Cells were also stained with DAPI, the DNA-binding fluorescent stain (blue). Each micrograph is representative of three independent experiments.

The MAP kinase signaling pathways have a well-documented role in cell proliferation and differentiation. Here, activation of the MAP kinase signaling pathways by PACAP-38 treatment of SH-SY5Y cells was examined. Cells maintained in low serum medium were stimulated for 15 min with a concentration range of 0.1 nmol/L to 1 μmol/L PACAP-38, and phosphorylation of ERK 1 and 2, p38 MAP kinase and JNK were assessed by western blotting using specific antibodies (Fig. 5). PACAP-38 stimulated phosphorylation of ERK (pERK), p38 MAP kinase (p-p38 MAP kinase) and JNK (pJNK) in a concentration-dependent manner (Fig. 5a), with EC_50_ values of 0.7 nmol/L, 1.5 nmol/L and 37.7 nmol/L, respectively. The pERK and p-p38 MAP kinase levels reached a maximum at 10–100 nmol/L PACAP-38, a similar concentration range required to obtain a maximum PACAP-differentiated phenotype. The increase in pJNK was more modest and required 10-fold greater concentration of PACAP-38 to reach a maximal level, compared to the other MAP kinases. Cells treated with 100 nmol/L PACAP-38 displayed prolonged phosphorylation of ERK and p38 MAP kinase (Fig. 5b) with pERK becoming localized in the nucleus after 30 min of stimulation (Fig. 5c). This is an event believed to switch ERK activity from that mediating proliferation to that inducing differentiation (Traverse *et al.* 1992; Ebisuya *et al.* 2005). Although the levels of pERK declined almost back to basal levels over the 2 h time course of the experiment, the levels of p-p38 MAP kinase remained elevated (Fig. 5b). JNK phosphorylation appeared weak and declined quickly in comparison.

**Fig. 6:**
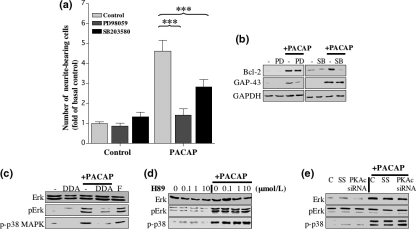
The effect of ERK and p38 MAP kinase on PACAP-induced differentiation of SH-SY5Y cells. (a–b) SH-SY5Y cells were cultured in low serum medium for 24 h and incubated with 10 μmol/L PD98059 (PD) or 10 μmol/L SB203580 (SB) for 45 min before treatment with 100 nmol/L PACAP-38 or low serum medium as control for 4 days. Treatments were replaced after the second day. (a) The fold increase in neurite-bearing cells was determined as described in Materials and methods. Standard error bars are shown (*n* = 3) and ANOVA was carried out to identify significant differences between treatments (***p < 0.001). (b) Western blot analysis of whole cell extracts using antibodies specific for Bcl-2 and GAP-43 was carried out on day 4. Protein loading was determined using an antibody specific for GAPDH. (c-e) Western blot analysis of whole cell extracts was carried out using antibodies specific for ERK, pERK and p-p38 MAP kinase. (c) Cells cultured in low serum medium for 24 h were incubated with 300 μmol/L DDA in the presence, or absence, of 100 nmol/L PACAP-38 or 10 μmol/L forskolin for 15 min. (d) Cells maintained in low serum medium for 24 h were treated with 0.1, 1 and 10 μmol/L H89 for 45 min, then with or without 100 nmol/L PACAP-38 for 15 min. (e) Expression of PKAc was inhibited by siRNA knock-down as described in Materials and methods (C = control, SS = scrambled siRNA sequence, PKAc siRNA = specific siRNA sequence targeting the catalytic subunit of PKA). Cells were incubated in the presence, or absence, of 100 nmol/L PACAP-38 for 15 minutes. Each blot is representative of three independent experiments.

Because of the strength and duration of PACAP-elicited phosphorylation of ERK and p38 MAP kinase, the involvement of these kinase pathways in PACAP-38-induced cell differentiation was examined. SH-SY5Y cells were maintained in low serum medium for 4 days with 10 μmol/L PD98059 (an inhibitor of MEK1), with 10 μmol/L SB203580 (an inhibitor of p38 MAP kinase) or in low serum medium alone as the control, with or without 100 nmol/L PACAP-38. Changes in cell morphology and expression of Bcl-2 and GAP-43 were determined. As shown in Fig. 6a, inhibition of the ERK pathway caused a significant reduction in the number of neurite-bearing cells induced by PACAP-38, from 4.6 ± 0.6-fold to 1.4 ± 0.4-fold of basal control, an inhibition of approximately 85% of the stimulated response. Inhibition of p38 MAP kinase pathway caused a more modest reduction in the number of neurite-bearing cells to 2.8 ± 0.4-fold of the basal control, an inhibition of approximately 58% of the stimulated response. The PACAP-induced increase in Bcl-2 levels was inhibited by SB203580 and not by PD98059 whereas the converse was true for GAP-43 (Fig. 6b).

The relationship between AC activation and MAP kinase activation was explored by incubating cells with 300 μmol/L DDA or low serum medium alone and stimulating with 100 nmol/L PACAP-38. The effects on the levels of phosphorylated MAP kinase following treatment with 10 μmol/L forskolin were examined as well. As shown in Fig. 6c, DDA inhibited the PACAP-38-stimulated increase in pERK and p-p38 MAP kinase. Forskolin, like PACAP-38, stimulates an increase in pERK and p-p38 MAP kinase. This was not prevented by inhibiting PKA, as neither H89 (Fig. 6d) nor siRNA-mediated knock-down of the catalytic subunit of PKA prevented the PACAP-38-induced increase in pERK and p-p38 MAP kinase levels (Fig. 6e).

### The Epac activator 8-CPT-Me-cAMP stimulates ERK but not p38 MAP kinase activity

**Fig. 7:**
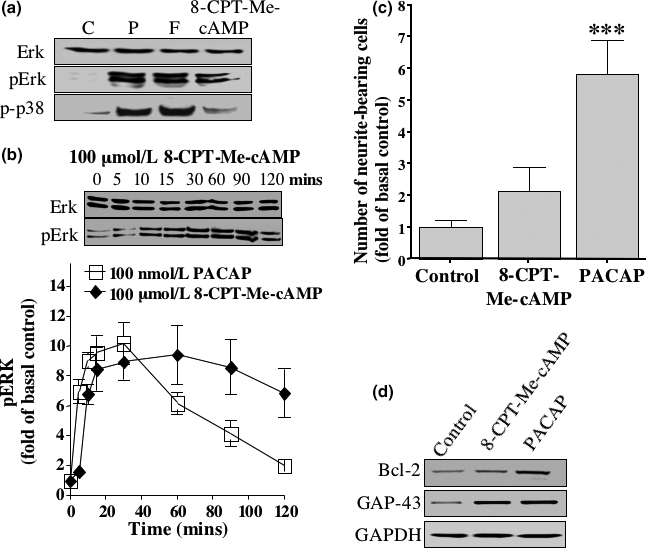
Involvement of Epac. (a) Western blot analysis of whole cell extracts was carried out using antibodies specific for ERK, pERK and p-p38 MAP kinase. The cells were maintained in low serum medium for 24 h and stimulated with the indicated factors for 15 min C = control, *p* = 100 nmol/L PACAP-38, F = 10 μmol/L forskolin and CPT-cAMP = 100 μmol/L 8-CPT-Me-cAMP. (b) Western blot analysis of whole cell extracts was carried out using antibodies specific for ERK and pERK. Cells were stimulated for up to 2 h with 100 μmol/L 8-CPT-Me-cAMP. The increase in phosphorylation levels was determined by densitometry as described in Materials and methods and is graphically represented in the lower section of the figure. Standard error bars are shown. (c) Cells were maintained in low serum medium for 24 h then stimulated with 100 μmol/L 8-CPT-Me-cAMP or with 100 nmol/L PACAP-38 for 4 days. Control cells were kept in low serum medium alone. The fold increase in neurite-bearing cells was determined as described in the Materials and methods. Standard error bars are shown (*n* = 3) and ANOVA analysis was carried out to identify significant differences between treatments (***p < 0.001). (e) Western blot analysis of whole cell extracts using antibodies specific for Bcl-2 and GAP-43 was carried out. Protein loading was determined using an antibody specific for GAPDH. Cells were treated for 4 days in low serum medium alone (Control) or treated with 100 μmol/L 8-CPT-Me-cAMP (CPT-cAMP) or with 100 nmol/L PACAP-38 (PACAP). Each blot is representative of three independent experiments.

The PACAP-38-stimulated activation of ERK and p38 MAP kinase is cAMP-dependent but PKA-independent in SH-SY5Y cells. The guanine nucleotide exchange factor, Epac, is also known to mediate cAMP-dependent activation of MAP kinase in certain cell types, including PC6 cells (Shi *et al.* 2006). We have examined this possibility here. Figure 7a shows the effect of the specific Epac activator, 8-CPT-Me-cAMP, PACAP-38 and forskolin on pERK and p-p38 MAP kinase levels. 8-CPT-Me-cAMP stimulated an increase in pERK but not p-p38 MAP kinase. The time course of this activation of ERK was examined and compared to that of 100 nmol/L PACAP-38 (Fig. 7b), cells treated with 100 μmol/L 8-CPT-Me-cAMP had similar levels of pERK to those treated with PACAP-38 within 30 min, but the levels declined more slowly than the PACAP-38-stimulated levels. However, 100 μmol/L 8-CPT-Me-cAMP elicited an increase in the number of neurite-bearing cells to 2.2 ± 0.9-fold of basal control compared to 5.8 ± 1.3-fold following treatment with 100 nmol/L PACAP-38 (Fig. 7c). Expression of GAP-43 but not Bcl-2 was up-regulated by 100 μmol/L 8-CPT-Me-cAMP (Fig. 7d).

## Discussion

Human SH-SY5Y cells differentiate into a more neuronal phenotype when treated with the differentiating factors RA, PMA, or brain-derived neurotrophic factor (Leli *et al.* 1992; Jamsa *et al.* 2004; Pan *et al.* 2005) and we have used these cells to explore the mechanisms involved in the neurotrophic actions of PACAP. This is the first study, to our knowledge, to examine the signaling pathways underlying PACAP-38-stimulated neuritogenesis in human cells. PACAP treatment of SH-SY5Y cells elicited a large increase in the number of neurite-bearing cells, corresponding to the formation of neurons during development and to their regeneration after injury. A number of studies have now identified PACAP as having a role in increasing axon growth and preventing cell death following: facial injury (Suarez *et al.* 2006); spinal cord injury (Kim *et al.* 2000); traumatic brain injury (Farkas *et al.* 2004); ischemia (Chen *et al.* 2006). The formation of growing axons is accompanied by an increase in expression of the growth cone protein, GAP-43 (Meiri *et al.* 1986): in SH-SY5Y cells, differentiating factors, such as RA and PMA have been shown to stimulate the expression of GAP-43 (Feng and Porter 1999). The anti-apoptotic protein Bcl-2 is also up-regulated following differentiation of SH-SY5Y cells (Feng and Porter 1999; Raguenez *et al.* 1999). In this study, we have shown that PACAP treatment causes a clear up-regulation of GAP-43 and Bcl-2 in SH-SY5Y cells, over the concentration range in which neuritogenesis is promoted (Fig. 1c). PACAP is known to regulate the formation of both noradrenergic and cholinergic neurons during development (May and Braas 1995; Braas and May 1996; Yuhara *et al.* 2003). Here we have shown that PACAP increases expression of the cholinergic marker ChAT, yet has little effect on the high basal expression of the noradrenergic marker, TH (Fig. 1c). PACAP therefore causes a switch from a noradrenergic to a mixed noradrenergic and cholinergic phenotype. These data show that PACAP is able to differentiate SH-SY5Y cells into a more neuronal phenotype, with the concomitant up-regulation of some well-characterized proteins involved in neuritogenesis and cell survival.

We have shown previously that the PAC_1_ receptor is primarily responsible for PACAP-elicited cAMP production in SH-SY5Y cells (Lutz *et al.* 2006). In this study, the use of the PAC_1_ receptor antagonist, PACAP6-38, in combination with the VPAC_2_ receptor-specific agonist, Ro 25-1553 has confirmed that the observed effects of PACAP on differentiation of SH-SY5Y cells are mediated by the PAC_1_ receptor (Fig. 2). Treatment of the cells with the AC inhibitor, DDA, and AC activator, forskolin, allowed us to determine that, as anticipated, cAMP is the primary mediator of PACAP-induced differentiation, influencing both neuritogenesis and the expression of both Bcl-2 and GAP-43 (Fig. 3).

It has been shown previously that neurite extensions are induced in SH-SY5Y cells by a cell permeable cAMP analogue, dibutyryl cAMP (db-cAMP) (Sánchez *et al.* 2004). However, there are important differences between our observations and those of the previous study regarding the involvement of PKA, ERK, and p38 MAP kinase in this process. Our study shows the strong activation of ERK and p38 MAP kinase following treatment with PACAP-38 and that this activation is dependent on AC activation. cAMP-mediated activation of ERK has been shown to be PKA-independent previously (Hashimoto *et al.* 2003), similarly, in this study, activation of ERK and p38 MAP kinase was not prevented by the PKA inhibitor, H89, or siRNA-mediated knock-down of PKAc expression. Furthermore, neuritogenesis was also observed to be cAMP-dependent but PKA-independent (Fig. 4b and c). Well-characterized inhibitors of ERK activation (PD98059) and p38 MAP kinase (SB203580) provided evidence that both ERK and p38 MAP kinase contribute to the differentiation of SH-SY5Y cells (Fig. 6a).

In the report by Sánchez *et al.* (2004) db-cAMP-induced neuritogenesis in SH-SY5Y cells involved a PKA-dependent but ERK- and p38 MAP kinase-independent mechanism. One explanation for the observed differences in results between the current study and that of Sánchez *et al.* is the differing cell culturing conditions used during the differentiation treatments, most notably the presence (this study) or absence of serum (Sánchez *et al.* 2004). It has been shown that culturing neuroblastoma cells, including SH-SY5Y cells, in serum-free medium induces neuritogenesis, even without the addition of differentiating agents (Alleaume *et al.* 2004; Héraud *et al.* 2004; Buttiglione *et al.* 2007). The effect of culturing SH-SY5Y cells in the serum-free medium without db-cAMP was not reported by Sánchez *et al.* (2004). However, this is not likely to be the only contributing factor. It has been reported that culturing SH-SY5Y cells in medium containing low levels of serum (1% or less) promotes neuritogenesis (Buttiglione *et al.* 2007). During the 4 day time course used in our study, we did not observe a change in the number of neurite-bearing cells when SH-SY5Y cells were maintained in low serum medium (containing 0.2% FBS). However, we deliberately used low passage cells (≤20) in our study as we observed that these did not spontaneously differentiate when moved into the low serum medium, whereas cells at higher passage numbers did (Monaghan and Lutz, unpublished observations). This suggests that SH-SY5Y cells may have regulatory mechanisms controlling differentiation processes that are lost at higher cell passage numbers, and that this should be considered when comparing results from different studies.

It seems likely that the different means of elevating intracellular cAMP levels that were used in the current study compared to the previous study (agonist-stimulated GPCR-mediated AC activation versus non-hydrolysable cAMP analogue) was a major determinant on which mechanism transduced the signal. It is generally agreed that cAMP-mediated signaling is very complex and from the large number of different reports, it seems possible that there is more than one cAMP-activated transduction pathway that will lead to neuritogenesis [see review by Dumaz and Marais (2005)]. It is becoming increasingly evident that compartmentalization and the oscillatory pattern of intracellular cAMP levels encode important information that is involved in determining cellular effects (Rich *et al.* 2001; Zaccolo and Pozzan 2003). Certainly, the neurite-guiding effect of PACAP has been attributed to this (Guirland *et al.* 2003). Indeed, when global cellular levels of cAMP were raised, this was blocked. Furthermore, the cAMP signal is often integrated with that of other signals, particularly Ca^2+^, that also regulate the activity of ACs and phosphodiesterases (Zaccolo and Pozzan 2003). The PAC_1_ receptor can also couple to the activation of phospholipase C and phospholipase D in addition to AC activation (McCulloch *et al.* 2001; Lutz *et al.* 2006), and the activation of these additional pathways may modify the cAMP signal and/or the resulting cellular effects evoked by PACAP. We are currently investigating this possibility. Maintaining cells for a long period of time in medium containing a constant amount of a stable cAMP analogue such as db-cAMP bypasses the GPCR/Gs/AC regulatory control mechanisms. Furthermore, the possible long-term global increase in intracellular cAMP levels may actually alter, or even prevent, the activation of pathways that mediate a more compartmentalized cAMP signal, such as may occur through GPCR-mediated activation of AC and presumably the subsequent regulation of the signal through the activation of phosphodiesterases, protein kinases and phosphatases that may be tethered or recruited into nearby signaling complexes. The role of cAMP compartmentalization in neuronal differentiation remains to be determined.

**Fig. 8:**
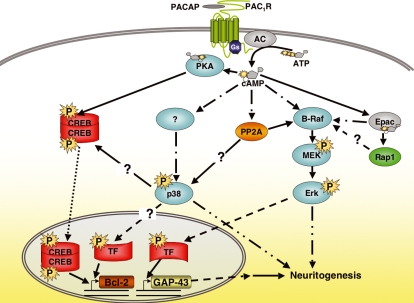
Proposed model for the cAMP-dependent signaling events involved in the neurotrophic actions of PACAP-38 on SH-SY5Y cells. PACAP-38 binding to the PAC_1_ receptor, which couples to the activation of AC, evokes an increase in the intracellular levels of cAMP (Lutz *et al.* 2006). This in turn leads to the activation of PKA-dependent and PKA-independent mechanisms that are involved in the induction of expression of neuronal proteins and in neuritogenesis, respectively. The PKA-dependent mechanism involves PKA phosphorylation of the transcription factor CREB, initiating its activation and translocation to the nucleus where it may be involved in the up-regulated expression of proteins involved in neuronal differentiation, including Bcl-2. The PKA-independent mechanisms involve activation of MEK and ERK as well as p38 MAP kinase that are required for neuritogenesis. The MEK/ERK activation possibly occurs through one or a combination of different mechanisms. The neuronal MAP kinase signaling cascade involves B-Raf as the first component (Dugan *et al.* 1999) and it has been shown that the 95 kDa B-Raf kinase is expressed in SH-SY5Y cells (Stephens *et al.* 1992). However, it is still unclear how cAMP activates B-Raf (Dumaz and Marais 2005). This may occur through activation of the cAMP-dependent Rap 1 GEF, Epac, or through another mechanism that may be potentiated by Epac (Lin *et al.* 2003) but may or may not involve Rap1 (Dumaz and Marais 2005). An additional possibility is the cAMP-mediated activation of PP2A (Feschenko *et al.* 2002) may directly induce B-Raf activation (Strack 2002). Activated ERK is required for neuritogenesis as well as being involved in the up-regulated expression of GAP-43, possibly through translocation to the nucleus. cAMP mediates the activation of p38 MAP kinase through a non-canonical pathway that may be similar to that observed in Th2 cells (Chen *et al.* 2000) and in cardiac fibroblasts (Yin *et al.* 2006), and which also may involve PP2A (Boudreau *et al.* 2004). Activated p38 MAP kinase in turn is involved in the increased expression of Bcl-2, possibly through phosphorylating CREB (Yin *et al.* 2006) and/or other factors, as well as in neuritogenesis. The arrows indicate direct interactions, the dotted arrows translocation, and the dot dash arrows the possibility of one or a number of intermediary steps that have not been worked out.

It is clear from studies of rat PC12 and related phaeochromocytoma cell lines that there are certain parallels in the mechanisms mediating the neurotrophic actions of PACAP on these cells [see review by Ravni *et al.* (2006)] and those which we have observed in SH-SY5Y cells. A role for Epac in the cAMP-mediated differentiation of neuronal cells has recently been described by Kiermayer *et al.* (2005), whose study in PC12 cells described a cAMP-mediated, PKA-independent activation of Epac causing a sustained activation of ERK activity resulting in the differentiation of neuronal cells. Shi *et al.* (2006) described distinct Epac/Rit/p38 and Epac/Rap/ERK pathways contributing to PACAP-38-stimulated neuritogenesis in another rat phaeochromocytoma cell line, PC6 cells. However, in the human cells, the specific activator of Epac, 8-CPT-Me-cAMP, caused a sustained activation of ERK, a clear increase in GAP-43 expression and a modest increase in neuritogenesis without influencing p38 MAP kinase activation (Fig. 7). PACAP-38 stimulated p38 MAP kinase through an AC-dependent and PKA-independent mechanism that does not appear to be Epac-mediated. The PKA-dependent activation of p38 MAP kinase has been described (Delghandi *et al.* 2005), in this case, however, PKA inhibition does not influence p38 MAP kinase phosphorylation or neuritogenesis. However, CREB phosphorylation and Bcl-2 expression are sensitive to inhibitors of both p38 MAP kinase and PKA, suggesting regulation by the coincident activation of these kinases. The activation of p38 MAP kinase is therefore cAMP-dependent but Epac- and PKA-independent (a non-canonical pathway recently described in Th2 cells (Chen *et al.* 2000) and mouse cardiac fibroblasts (Yin *et al.* 2006)). These data therefore indicate that the PKA-independent, Epac-mediated activation of ERK and cAMP-dependent, Epac-independent activation of p38 MAP kinase pathways takes place in response to PACAP-38 treatment and that both these pathways contribute to PACAP-38-stimulated neuritogenesis in human neuroblastoma cells. Interestingly, the cAMP-dependent but PKA-independent mechanism involved in the activation of ERK in PC12 cells has not been entirely elucidated and there is the distinct possibility that there may be additional novel cAMP-regulated components involved in this (Gerdin and Eiden 2007) as appears to be the case in SH-SY5Y cells. One possibility that we are investigating is the cAMP-mediated activation of protein phosphatase 2A (Feschenko *et al.* 2002) may induce B-Raf activation (Strack 2002) as well as p38 MAP kinase activation (Boudreau *et al.* 2004). A proposed model of the signaling pathways involved in mediating the neurotrophic actions of PACAP in SH-SY5Y cells is shown in Fig. 8.

In summary, we have shown that PACAP-38-mediated differentiation of SH-SY5Y cells is cAMP-dependent and involves both PKA-dependent and PKA-independent mechanisms. The increase in cAMP levels led to phosphorylation of ERK, p38 MAP kinase and JNK via a PKA-independent mechanism. Activation of AC, ERK and p38 MAP kinase were shown to be essential to fully differentiate SH-SY5Y cells with PACAP. The cAMP-dependent guanine nucleotide exchange factor, Epac, was implicated in the activation of ERK and the resultant neuritogenesis. These results provide further evidence of the neurotrophic actions of PACAP and provide insight into how PACAP may function during neuronal development and regeneration, highlighting the importance of cAMP, Epac, ERK, and p38 MAP kinase in these processes.

## Abbreviations used

AC: adenylyl cyclase
ChAT: choline acetyltransferase
CREB: cAMP response element binding protein
DDA: 2′,5′-dideoxyadenosine
Epac: exchange protein directly activated by cAMP
ERK: extracellular signal-regulated kinase
FBS: fetal bovine serum
GAP-43: growth-associated protein-43
GAPDH: glyceraldeyhde-3-phosphate dehydrogenase
GPCR: G protein-coupled receptor
JNK: c-Jun N-terminal kinase
MAPK: mitogen-activated protein kinase
PAC_1_: PACAP receptor type I
PACAP: pituitary adenylate cyclase-activating polypeptide
PKA: protein kinase A
PLC: phospholipase C
PMA: phorbol 12-myristate 13-acetate
PP2A: protein phosphatase 2A
RA: retinoic acid
TH: tyrosine hydroxylase
VIP: vasoactive intestinal peptide
VPAC_1_: VIP/PACAP receptor type I
VPAC_2_: VIP/PACAP receptor type 2
